# The Modified Hospital Elder Life Program (HELP) in geriatric hospitalized patients in internal wards: A double-blind randomized control trial

**DOI:** 10.1186/s12877-021-02520-3

**Published:** 2021-10-26

**Authors:** A. Kojaie-Bidgoli, F. Sharifi, F. Maghsoud, M. Alizadeh-Khoei, F. Jafari, F. Sadeghi

**Affiliations:** 1 Department of Gerontology, University of Social Welfare and Rehabilitation Sciences, Tehran, Iran; 2 Elderly Health Research Center, Endocrinology and Metabolism Population Sciences Institute, Tehran University of Medical Sciences, Tehran, Iran; 3 Department of Medical-Surgical Nursing, Faculty of Nursing and Midwifery, Kashan University of Medical Sciences, Kashan, Iran; 4 Clinical Gerontology & Geriatric Department, Medical School, Tehran University of Medical Sciences, Tehran, Iran; 5 Department of Psychiatric Nursing, Faculty of Nursing and Midwifery, Kashan University of Medical Sciences, Kashan, Iran

**Keywords:** Elderly, Patients, Delirium, Prevention, Geriatric, Hospital elder life program

## Abstract

**Background:**

Hospital Elder Life Program (HELP) provides protocols based on factors for reducing delirium. Due to the lack of geriatric wards and aged care teams in Iran, it seems that some of the original HELP interventions need to be modified through a trial study. Hence, this study was conducted to determine whether the Iranian modified HELP could reduce delirium in geriatric hospitalized patients.

**Methods:**

This double-blind randomized controlled trial was designed and conducted in a hospital at Kashan University of Medical Sciences in Iran. A total of 195 hospitalized patients aged ≥70 years, were 84 in the Intervention Group (IG) and 111 in the Control Group (CG). After assessing delirium risk factors, participants in the IG group received interventions based on the cognitive, vision/hearing, sleep, mobility, feeding, and hydration protocols by nursing students and the CG group received routine care.

Delirium incidence was assessed by the Confusion Assessment Method. Delirium incidence, cognitive and functional abilities, frailty, fall, and length of stay were outcomes.

**Results:**

The mean age of the patients was 78.53(Standard Deviation = 5.87) years. Delirium incidence was higher in the CG comparing to IG (14.71% vs 3.66%).Significant reduction observed in risk incidence of delirium because of interventions [Odds Ratio:0.124, Confidence Interval: 0.03–0.48].

**Conclusion:**

The modified HELP effectively reduced delirium rates in geriatric hospitalized patients.

**Trial registration:**

This study was registered at the Iranian Registry of Clinical Trials IRCT20180910040995N1.

## Background

Increasing the Iranian elderly population from 6.4% in 2020 to 11% in 2036 is inevitable [1]. Older people are at a higher risk of hospitalization [2]. The prolonged hospitalization of elderly patients reduces their cognitive and functional abilities [3].

Delirium is a common geriatric syndrome that affects one-third of the hospitalized elderly patients [4]. Delirium has adverse effects on the patients’ recovery, functional abilities, length of hospital stay, admission in long-term centers, and rates of death [5, 6]. Standard treatments under medical conditions may be difficult due to the development of delirium [7]; so, primary prevention could be the most efficient strategy to decrease delirium, since at least 30 to 40% of delirium cases are preventable [8, 9] and the prevention of delirium is preferred to its treatment [10].

Implementing the multicomponent interventions’ with help of an interdisciplinary aged care team could be an effective strategy to prevent delirium; because the members of aged care teams recognize the multifactorial etiology of delirium [11].

The Hospital Elder Life Program (HELP) is a multi-component intervention for dealing with risk factors in delirium (reversible cognitive impairment, sleep deprivation, immobility, visual impairment, hearing impairment, and dehydration) in the hospital settings, implemented by interdisciplinary teams, included a geriatric nurse, elder life specialists, trained volunteers, and geriatricians who work closely with the primary nurses [12]. The HELP program is effective in preventing delirium [8, 13, 14], cognitive and functional decline [15], fall in hospital [13, 16], and decreasing in the length of hospital stay [13, 14, 17]. The program is cost-effective and reduces hospital costs [17, 18]. It has been shown that HELP is an effective program and can reduce delirium rates by 40% [8] and it can also reducethe rates of functional decline in hospitalized older patients by 67% [15].

The literature reveals that today, hospitals have made efforts to use the HELP program, but a lack of aged care services for hospitalized elderly in Iran, and deficiency of geriatric wards in Iranian hospitals made preventing the integration of HELP into medical care system. Consequently, it is necessary to carry out a trial study to modify some interventions and protocols in the HELP program which are compatible with the Iranian facility, care system capability, and the nature of the Iranian elderly population. Meanwhile, some interventions, which depend on the patients’ literacy, should be changed due to the high illiteracy rates in the Iranian elderly population [19]. Moreover, in the Iranian culture, the family members support the elderly and take care of these patients even in hospital settings [20], so, some interventions, which depend on the patients’ families, have to be modified in the HELP model. Besides, in the Iranian care system, getting help from volunteers in hospitals is not common; therefore, there is a need to change the volunteers’ protocols in the Iranian HELP version. Accordingly, it seems that it is necessary to modify the HELP program in developing countries based on their potential and abilities.

Therefore, the present study was conducted to determine the effect of the Iranian modified HELP on the prevention of delirium among hospitalized elderly patients in internal wards, due to the rapid growth of the Iranian elderly population, also the high prevalence of delirium (22%) among the Iranian hospitalized elderly patients, lack of adjustability of the original HELP program with the present Iranian hospital facilities, and a lack of studies on the modified HELP program in developing countries [21]. Besides, this study made an effort to assess the effects of HELP on the other program outcomes including the prevention of frailty, recovery from physical function and cognitive function disabilities, the decrease in fall rates in hospitalized patients, and the reduction in the length of hospitalization.

## Method

### Study design

On this study used a parallel-group, double-blind (evaluation and analysis), randomized clinical trial that was designed based on recommendations to conduct trial studies [22]. The participants were selected using the allocation stratified block random sampling method. This study was registered at the Iranian Registry of Clinical Trials IRCT20180910040995N1 at 2019-02-07.

### Study population

The participants, who comprised geriatric patients, were selected from the Internal Medicine wards of a university hospital in Kashan province of Iran for a period that lasted from October 2019 to October 2020. The inclusion criteria were as follows: being 70 years old and over, being admitted into one of the Internal Medicine wards, not being delirious at admission time, having at least one of the delirium risk factors at the time of admission (cognitive impairment, vision/hearing impairments, immobilization, sleep deprivation, dehydration: BUN/Cr ratio > 18), being willing to participate in the study, and being able to communicate verbally or in writing.

The exclusion criteria included coma, mechanical ventilation, aphasia (expressive and/or receptive), severely impaired communication ability, terminal/end stage conditions, imminent death, combative or dangerous behaviors, a severe psychotic disorder that prevent patients from participating in interventions, severe dementia (being unable to communicate based on SPMSQ 10 errors), airborne precautions (e.g., tuberculosis), being isolated, droplet precautions (e.g., influenza), neutropenic precautions, being discharged around 48 h after admission, patient’s refusal to participate in the study, and patient’s family members or physician’s refusal to let the patient participate in the study in the case of incompetent patients.

### Interventions

Based on the results of admission into the hospital, the participants in the intervention group received HELP interventions for 5 days with the help of three volunteer nursing BSc students. On the other hand, the participants in the control group received usual medical and nursing care during the hospitalization process.

The volunteers identified the principal family caregivers within the first 24 h of the admission and used a face-to-face communication strategy to train them to provide the patients with some interventions. Moreover, the volunteer nurses delivered them with an informative booklet, which contained information on certain strategies and recommendations to prevent delirium. Furthermore, they provided the family caregivers with a notebook and pencil for two reasons: one to record the conditions in which the interventions were provided to the patients and two to mention the non-adherence reasons in the conditions in which they did not provide the patients with the relevant interventions. Interventions were followed according to HELP protocols. They were translated into the Persian language and modified according to the Iranian culture, accessibility to facilities, potential of services, and types of equipment in this pilot hospital like as Focus Group Discussions (FGD) among head nurses and nursing supervisors. Then the modified interventions were organized based on FGD meetings and HELP protocols. Finally, they were modified, based on the daily visits of the team director.

The protocols were included; cognitive protocol, vision/ hearing protocol, sleep protocol, mobility, hydration and feeding assistance.

#### Cognitive protocol

Orientation (patient orientation card, including names of care team members, time, place, and daily schedule), cognitive stimulation, therapeutic activities (discussion of current events, puzzle, and memory games). All of the interventions provided are based on the cognitive - HELP protocol.

#### Vision/ hearing protocol

Reminding the patients on using their glasses or hearing aids, training caregivers to communicate with the patients suffering from vision/hearing impairment. Some activities were unsuccessful, due to the lack of facilities including magnifying lenses and adaptive equipment (e.g., large illuminated telephone keypads, large print books, and fluorescent tape on the call bell), lack of daily reinforcement of the portable amplifying. Also, the patients’ referral to the specialists for ear wax removal. Nonetheless, the caregivers were given some training on how to administer vision/hearing interventions based on the HELP protocol.

#### Sleep protocol

The suggested sleep improvement interventions based on the original HELP included: using individual considerations for normal routines (can you think about something that might help you to go to sleep, that is, something you did not at home when you had trouble sleeping), making offer some interventions to the patient and caregiver (warm milk, back-rub, relaxation music play on a portable music player), doing additional sleep-promoting activities (avoiding caffeine after 2 p.m., exercising during the day as much as possible, avoiding day time napping, maintaining a regular time for going to bed each night), using strategies to reduce the noise in the wards (e.g., silent pill crushers, vibrating beepers, and quiet hallways), and adjusting schedules to facilitate sleep uninterrupted (e.g., re-scheduling the medications and procedures). Although most interventions were informed to the patients and caregivers, some interventions (drinking herbal tea, relaxing with music, back massage, using silent pill crushers, and vibrating beepers) could not be provided. Likewise, the nurses were trained to use some strategies including noise reduction in the wards, and re-scheduling in medications and procedures.

#### Mobility protocol

All of the suggested interventions were provided to the patients based on the original HELP, including ambulating or doing active range of motion exercises three times daily, minimizing the use of immobilizing equipment (e.g., bladder catheters and restraints). Moreover, several amenities (cane and walker in the pilot wards) were provided to the patients.

#### Feeding assistance protocol

The feeding assistance and encouragement during the meal followed based on the original HELP protocol.

#### Hydration protocol

Fluid repletion interventions (an early recognition of dehydration and oral volume repletion, i.e., encouragement of oral intake of fluids) was followed according to the hydration - HELP protocol.

Table 1 provides a comparison of the original HELP protocols and adapted HELP to the Iranian situation.

**Table 1:** Comparison between the original and the Iranian HELP programs

	Original HELP protocols	Implementing HELP in this study
**Screening**	Elder life nurse specialist within 48 h	Geriatric nurse specialist within 48 h
**Exclusion criteria**	Intubation or respiratory isolation, aphasia, terminally ill, severe dementia, respiratory isolation, and expected discharge within 48 h after admission.	Another exclusion criterion was added (admitting in the participants’ group wards for the second time).
**Protocols**	Orientation/daily visiting: Orienting the board with the names of care team members, daily schedule, and orienting communication.	All interventions were done, without changes.
	Therapeutic activities: Cognitive stimulation activities three times daily (e.g., discussion of current events, structured reminiscence, and word games).	Both of them were done as well as telling the story.
	Sleep enhancements: Individualized considering of normal routines (can you think of something that might help you to sleep, or you did at home when you had trouble in sleep), offering to the patient and caregiver (drinking warm milk, back-rub, relaxation with a portable music player), additional sleep-promoting actions (avoiding caffeine after 2 p.m., increase exercise and mobility during the day as much as possible, avoid daytime napping, preserve regular time for going to bed each night), ward-wide noise reduction strategies (e.g., silent pill crushers, vibrating beepers, and quiet hallways), and schedule adjustments to allow uninterrupted sleep (e.g., re-scheduling of medications and procedures).	Some interventions were not provided, e.g., drinking herbal tea, relaxing with music, back massage, using silent pill crushers, and vibrating beepers. Although, most intervention strategies’ informed the patients and caregivers. Also, noise reduction strategies were trained by patients and caregivers. The nurses were trained about ward-wide noise reduction strategies in the re-scheduling of medications and procedures.
	Early mobilization: Ambulating or active range-of-motion exercises three times daily and minimizing the use of immobilizing equipment (e.g., bladder catheters, restraints).	All interventions were done, without changes.
	Vision protocol: Visual aids (e.g., glasses or magnifying lenses) and adaptive equipment (e.g., large illuminated telephone keypads, large print books, and fluorescent tape on call bell), with daily reinforcement of their use.	Reminding in use of own glasses, caregivers’ training in how to communicate with the patient with the vision impairment. These activities failed, because of lacking facilities included magnifying lenses and adaptive equipment (e.g., large illuminated telephone keypads, large print books, and fluorescent tape on call bell)
	Feeding Assistance: Feeding assistance and encouragement during meals	All interventions were done, without changes.
	Fluid repletion: Early recognition of dehydration and oral volume depletion, i.e., encouragement of oral intake of fluid	All interventions were done, without changes.
**volunteers**	Volunteer shifts: Ranging from one to three times daily based protocols. Role of the volunteers: Providing program interventions, directly at the bedside.	Daily, once in the morning or the evening. Volunteer duty: Teaching family members and supervising them during the provision of the HELP interventions.
**Nursing staff**	The ELS and ELNS are in contact with the staff nurses.	The program director, geriatric nurse, and volunteers were in contact with the staff nurses.

### Study variables

Within the first 48 h of admission, the elderly patients were evaluated by researchers based on the inclusion criteria. After asking the patients or their family members to sign the consent form and getting their permission to involve the patients in the study, the patients were randomly placed in one of the HELP or control groups. The selected patients were evaluated by three geriatric nurses. The gathered data included:

Sociodemographic data (age, sex, marital status, education level, accommodation, type of insurance, monthly income level), polypharmacy, addiction, smoking, alcohol abuse, support by a family member, use of walking aids, health status, oral health, level of ambulation, hospitalization history, past medical history, ability to move and walk, mobility level (independent or needs assistance), and the ability to climb stairs.

Impairments in the basic Activities of Daily Living (ADL) before hospitalization was assessed using the Barthel index, which measures the elderly patients’ functional abilities during the performance of 10 activities in daily life. The internal consistency of the Iranian version BI was significant (Cronbach’s alpha = 0.938, *P* < 0.001) [23].

The Lawton Instrumental Activity of Daily Living (IADL) scale was used to assess the status of elderly patients before hospitalization. The sensitivity and specificity of the Iranian version of Lawton IADL were reported to be 0.75 and 0.96, respectively, the Cronbach alpha and ICC were higher than 0.75 [24].

The patients’ cognitive impairment was determined using the Pfeiffer’s Short Portable Mental Status Questionnaire (SPMSQ), the reliability was determined to be 0.88 with a cut-off point three for Iranian older adults [24].

Moreover, the Digit Span (DS) was used to assess the patients’ short-term memory and attention, as a test of attention and working memory [25]. At cut-off < 3 digits, the sensitivity and specificity of DSB in the detection of major cognitive disorders (including dementia, delirium, and cognitive impairment which are not otherwise specified) obtained 77 and 78%, respectively [26].

Frailty was assessed using the Rockwood Frailty Index. This was derived from the Canadian Study of Health and Aging (CASH), which was highly correlated (*r* = 0.80) with the Frailty Index [27].

Delirium was assessed using the Confusion Assessment Method (CAM) scale [8]. In an Iranian study, the sensitivity and specificity of the ICU CAM scale were reported being 66.7 and 99.1%, respectively [26].

The other assessment tools at the admission time were the Charlson Comorbidity Index [28], dehydration index, the number of medications that were prescribed, visual acuity, hearing impairment, nutrition status, and sleep.

Moreover, medical or nursing procedures (bladder catheterization, nasogastric tube placement, venous or arterial access, blood sample acquisition), and other invasive procedures were recorded.

### Outcomes

The primary outcome was to reduce the incidence of delirium in hospitalized elderly in Internal Medicine wards, which was assessed by the Confusion Assessment Method (CAM) scale [29]. Nurses performed a daily delirium evaluation with CAM and recorded it in every shift, also a well-trained nurse examined the patients on the incidence of daily delirium by interviewing their caregivers. The secondary outcomes included changes in physical function status, cognitive function, frailty, and dehydration between admission (T0) and hospital discharge (T1) times, also the length of hospital stay (LOS), and fall incidence in hospital.

### Sample size

A sample of 46 elderly patients in each group was selected using the 3.192 version of G*Power software and x^2^ test, and considering the effect size 0.6, alpha =5%, and beta =10%. The sample size was considered to be 55 patients per group, due to a 20% difference. Finally, this study involved 84 geriatric patients in the intervention group and 111 elderly patients in the control group.

### Randomization

We used stratified sampling since delirium risk (moderate and severe level) confounds the results. The elderly participants were randomly assigned to two groups using a simple random sampling method, used by six blocks with a proportion of 4: 2.

### Blinding

This study used the double-blind method. The patients and people who assessed the patients were uninformed about the objectives of the study and the patients’ condition in the intervention group and control group, although, the researcher was informed about them. Several measures were used to prevent communication between these groups. These measures included selecting only one patient per room (all rooms had four beds) so that other patients and their caregivers could not observe the implemented interventions. Interventions and evaluations were carried out by different personnel to avoid measurement biases. Moreover, the personnel were trained in separate sessions and the data were analyzed by a person who had not taken part in the evaluation and intervention processes.

### Help team

In the original HELP manuals, an Elder Life Specialist (ELS) and an Elder Life Nurse Specialist were involved in the team [11]. While, in the Iranian HELP team was applied a gerontologist (PhD) as the director of the program, three geriatric nurses, and three nursing students as volunteers. A team director planned the interventions, according to the delirium risk factors that were detected with the used tools in HELP. Some interventions were changed based on the daily evaluation of the elderly patients by a team director.

### Training and applying volunteer

Volunteers were selected from the BSc nursing students, who were interested in taking care of elderly patients. They were trained by a director of the program. Volunteers’ training consisted of classroom instructions including didactic training and small groups demonstration. At the end of the training sessions, the researchers administered a test to the volunteers based on the volunteers’ manual. Next, the volunteers provided the interventions according to the plan that the team director organized, then the director recorded all of the interventions in the patients’ files.

### Statistical methods

Statistical significance was appointed at *p* = 0.05. All of the tests were two-tailed tests. A descriptive analysis of the study variables was carried out (means, standard deviations, number of cases, and proportions). The normality of distribution was checked using the Shapiro–Wilk test. The researchers used both parametric statistics(i.e., Student’s t-test) and nonparametric statistics (i.e. Man-Whitney test) to analyze the collected data. The efficacy of the intervention was examined using the relative risk ratio (RR) and 95% confidence interval (95% CI). All of the analyses were performed as an intention to treat approach. The data analysis was carried out using SPSS20 statistical package.

### Ethical aspects

After getting permission from Dr. Sharon Inouye, who is the developer of the original HELP, the contents and protocols of the program were separately translated into Persian, with the help of two translators and one gerontologist. Moreover, the contents of the program were modified, based on the Iranian hospital care system. The Research Ethics Committee of the University of Social Welfare and Rehabilitation Sciences approved this study (IR.USWR.REC.2017.5.25). In terms of ethical considerations, first, the aims of this study were explained to the participants and their caregivers. Next, informed consent was obtained from illiterate participants and their family members.

## Results

**Fig. 1:**
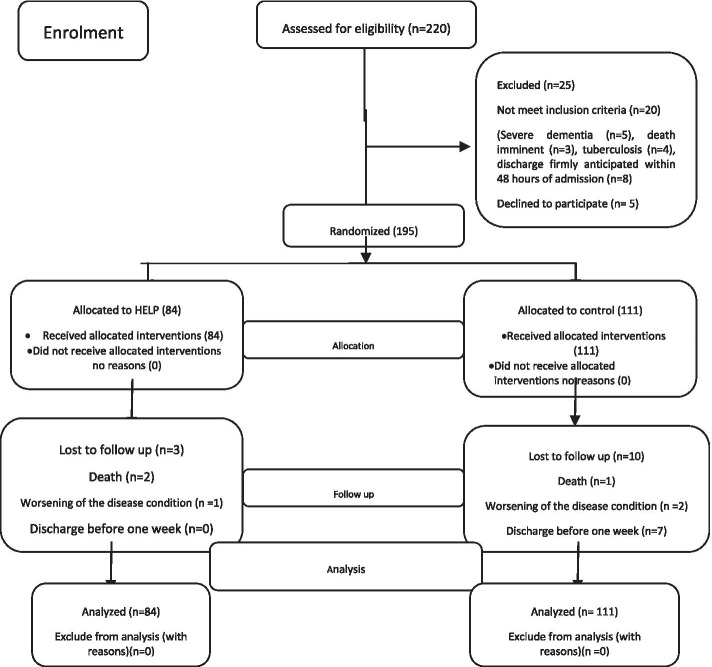
The participants’ flow diagram (CONSORT 2010) [30]

In this study, 220 geriatric patients were evaluated for eligibility. Based on the results of the evaluation,195 elderly patients met the inclusion criteria (88.6%). Regarding the objectives of this study, 84 elderly patients (43.07%) from among the 195 eligible patients were randomly assigned to the intervention group and 111 (56.92%) of them were assigned to the control group. Two participants in the intervention group (2.4%) and one patient in the control group (0.9%) died during hospitalization. Nonetheless, the collected data on delirium status were available for these subjects. Moreover, one patient in the intervention group (1.2%) and two patients in the control group (1.8%) can not continue the study, due to the worsening condition of the disease. Moreover, seven patients in the control group (6.3%) were excluded from the study, since they were discharged from the hospital before 1 week (Fig. 1).

The mean ages of the participants in the intervention and control groups were 79.12 (SD: 5.72) and 77.75 (SD: 6.01) years, respectively. In both of the groups, most of the patients were male (intervention group: 59.5%, control group: 55.9%). More than half of the participants (62.56%) were illiterate (interventiongroup: 57.14%, control group: 66.67%).The other characteristics of the geriatric patients in each group at admission time are provided in Table 2. There were not any significant differences between the intervention group and the control group in terms of any of the characteristics. The mean number of the risk factors per patient at admission time was similar in both groups [Table 2].

**Table 2:** Characteristics of the geriatric hospitalized patients on admission time

Variables	Complete sample	Control group	HELP group	*P*-value
Gender(n) %				0.608
Male	(112) 55.9%	(62)55.9%	(50) 59.5	
Marital Status				0.276
Married	(129) 66.15%	68(61.26%)	(61)72.62%	
Widowed	(63) 32.31%	40(36.4%)	(23)27.38%	
Other	(3) 1.53%	3(0.27%)	0	
Education				0.176
Illiterate	(122)62.56%	(74)66.67%	(48)57.14%	
Primary School	(65)33.33%	(35)31.53%	(30)35.71%	
High School	(2)2.38%	0	(2)1.03%	
University	(6)3.08%	(2)1.8%	(4)4.76%	
Polypharmacy				0.647
Less than three drugs	(52)26.67%	(31)27.93%	(21)25%	
More than three drugs	(143)73.33%	(80)72.07%	(63)65%	
AddictionYes	(14)7.18%	(10)9.01%	(4)4.76%	0.255
Charlson Comorbidity Index; mean ± SD	2.72 ± 1.83	2.66 ± 1.82	2.79 ± 1.85	0.624
Hypertension	(118)60.51%	(67)60.36%	(51)60.76%	1.000
Diabetes	(79)40.51%	(38)34.23%	(41)48.81%	0.055
^a^CAD	(103)52.82%	(57)51.35	(46)54.76	0.834
CHF^b^	(27)13.85%	(18)16.22%	(9)10.71%	0.271
^c^CVA	(14)7.18%	(8)7.21%	(6)7.14%	1.000
Parkinson	(4)2.055	(3)2.70%	(1)1.19%	0.636
History of dementia	(18)9.23%	(12)10.81%	(6)7.145	0.381
Kidney failure	(31)15.90%	(18)16.22%	(13)15.48%	0.889
Liver failure	(7)3.59%	(5)4.50%	(2)2.38%	0.701
COPD	(28)10.81%	(12)10.81%	16)19.05%)	0.148
Depression	(15)7.69%	(7)6.31%	(8)9.53%	0.461
Cancer	(8)4.10%	(5)4.50%	(3)3.57%	1.000
Frailty Index				0.191
Very fit	(1)0.5%	0	(1)1.2%	
Well – without active disease	(14)7.2%	(7)6.3%	(7)8.3%	
Well, with treated comorbid disease	(31)15.9%	(20)18%	(11)13.1%	
Apparently vulnerable	(52)26.7%	(30)27%	(22)26.2%	
Mildly frail	(24)12.3%	(9)8.1%	(15)17.9%	
Moderately frail,	(53)27.2%	(30)27%	(23) 27.4%	
Severely frail	(20)10.3%	(15)13.5%	(5)6%	
Age; mean ± SD (years)	5.87 ± 78.53	6.01 ± 77.75	5.72 ± 79.12	0.108
SPMSQ score mean ± SD	3.30 ± 2.96	3.50 ± 3.12	2.73 ± 3.05	0.288
score Digit span mean ± SD	4.76 ± 2.28	4.20 ± 2.85	5.19 ± 2.81	0.229
ADL-Barthel mean ± SD	16.46 ± 5.37	16.18 ± 5.29	16.83 ± 5.48	0.015
Targeted risk factors				
Cognition impairment	(102)52.2%	(59)53.2%	(43)51.2%	0.217
Immobility	(114)58.46%	(66)59.41%	(48)57.14	0.255
Visual impairment	(126)64.26%	(73)65.77%	(53)63.10%	0.534
Hearing impairment	(78)40%	(48)43.24%	(30)35.71%	0.334
Dehydration	(79)40.5%	(41)36.9%	(38)45.2%	0.242
Sleep disorder	(88)45.13%	(47)42.34%	(41)48.81%	0.273

^a^Coronary Arty Disease

^b^Chronic Heart Failure

^c^CerebroVascular Accident

Table 3 provides the primary and secondary outcomes during hospitalization. The primary outcomes showed that 18 (9.78%) participants had delirium during hospitalization.

**Table 3:** HELP-related outcomes during hospitalization in study groups

Outcomes	Total sample (*n* = 184)	Control group (*n* = 102)	HELP group (*n* =82)	*P*-value
Delirium (yes/no)	18(9.78%)	15(14.71%)	3(3.66%)	0.003
Frailty				
Very fit	(1)0.5%	0	(1)1.2%	< 0.001
Well – without active disease	(15)8.2%	(4)3.9%	(11)13.6%	
Well, with treated comorbid disease	(28)15.3%	(19)18.6%	(9)11.1%	
Apparently vulnerable	(60)32.8%	(19)28.4%	(31)38.3%	
Mildly frail	(19)10.4%	(5)4.9%	(14)17.3%	
Moderately frail	(40)21.9%	(30)29.4%	(10)12.3%	
Severely frail	(20)10.9%	(15)14.7%	(5)6.2%	
fall (yes/no)	(6).3.3%	(4)4%	(2)2.5%	0.694
length of stay in hospital	8.02 ± 3.52	7.62 ± 1.49	8.00 ± 2.24	0.196
SPMSQ score	4.65 ± 1.48	3.68 ± 2.74	2.65 ± 2.49	0.009
score Digit span	4.63 ± 2.86	4.19 ± 2.84	5.18 ± 2.81	0.021
ADL-Barthel score	16.01 ± 5.24	15.18 ± 5.59	17.04 ± 4.59	0.015
Dehydration	(73)40.1%	(36)35.6%	(37)45.7%	0.170

The delirium incidence rate was higher in the control group (14.71%) in comparison with the interventiongroup (3.66%) (Odds Ratio [OR] 0.12), *P* = 0.003). Logistic regression results (after entering age, sex, and frailty variables) showed a statistically significant reduction in the risk of delirium due to the intervention. The results of the logistic regression showed that frailty increased the incidence of delirium (OR: 1.8, CI: 1.16–3.08).

According to the Rockwood Frailty Index, at admission time, most of the patients in the interventiongroup (27.4%) and the control group (27%) were at the sixth level of frailty (moderately frail) and there were not any statically significant differences between these groups in this regard. At discharge time, most of the patients in the interventiongroup (38.3%) were at the fourth level of the Rockwood Frailty Index (apparently vulnerable). On theother hand, in the control group, most of the patients were at the sixth level (29.4%) of frailty and there were statically significant differences between the two groups regarding the frailty level (*P* < 0.001). The results of the logistic regression (after entering age, sex, delirium, and frailty at the admission variables) indicated a statistically significant reduction in the risk of frailty due to the intervention (OR: 0.124, CI: 0.03–0.48). Besides, statically meaningful difference in age (OR: 1.1, CI: 1.002–1.022), delirium (OR: 22.76, CI: 2.10–246.09), and being frail in the admission time (OR: 111.55, CI: 27.14–458.41) observed with frailty indischarge time.

Furthermore, the rates of fall in the control group (4%) were higher than the fall rates in the HELP group (% 2.5). Nonetheless, no significant difference was found between the groups concerning the patients’ fall rates.

In the selected sample, the mean length of hospitalization was 8.02 days (SD = 3.52). In the intervention group, the length of hospitalization was higher than that of the control group. However, this difference between the groups did not reach the significance level.

At the admission time, the differences in the scores on SPMSQ were not statistically significant. Notwithstanding, at the discharge time, the mean score of the incorrect answers on SPMSQ in the intervention group was lower than the mean score of these answers on SPMSQ in the control group and this difference reached the significance level (2.65 vs 3.68, diff mean: 1.02, *P* = 0.009).

At the admission time, the differences in scores on the Digit Span were not statistically significant. However,the mean value of Digit Span in the interventiongroup was more than the mean value of the answers to this test in the control group. At the discharge time, while the mean score of Digit Span increased in the interventiongroup, it decreased in the control group and statistically significant differences were observed between the intervention group and control group regarding the mean value of Digit Span (5.1 vs 4.1, mean diff: 0.987, *P* = 0.02).

Concerning the functional measure, the participants in the intervention group had a higher mean value in ADL-Barthel in comparison with the participants in the control group at the discharge time. Furthermore, a significant difference was observed between the two groups (17.04 (SD: 4.59) vs 15.18 (SD: 5.59), mean diff: 1.86, *P* = 0.01). However, the mean difference between the groups on ADL-Barthel at admission and discharge times were not statistically significant.

## Discussion

This paper summarizes an example of the successful modification and implementation of a clinical program (HELP). Regarding the feasibility of the Iranian HELP version, the results of the present study showed that it was possible to implement this model in Iran, despite cultural differences and limited human resources.

This study indicated that the Hospital Elder Life Program (HELP) is effective in preventing delirium in hospitalized geriatric patients. Moreover, it showed that some modified interventions in the original HELP program could prevent the incidence of delirium.

Moreover, our findings showed that the HELP program could recover the frailty syndrome in participated patients in HELP. Chen et al. [31] reported that modified HELP on gastrointestinal surgery older patients decreased the rates of frailty incidence during hospitalization in the HELP group.

Moreover, our findings indicated that the Iranian HELP version was affective to protect in cognitive decline. In a study, Huson et al. [32] showed the effectiveness of the HELP program for the elderly patients ≥70 years old who were admitted to a rehabilitation setting and reported that the patients who received the HELP showed greater improvement regarding the cognitive and functional outcomes including short-term memory and recall. In the present study, one of the effective factors which improved the patients’ cognitive abilities was the regular presence of a member of the patient’s family in the patient’s room to provide the patient orientation/ therapeutic activity interventions in the cognitive protocol.

Our findings showed that the HELP interventions improved functional abilities among elderly patients in the hospital. In our study, the patients, who received 7 days of HELP interventions, had a one-point increase in ADL –Barthel score.

Likewise, a study on the functional benefits of the HELP program reported that the ADL function (assessed by the BI) in the control group decreased by 27.9 points during 2 weeks of hospitalization. However, it decreased by only 11.8 points in the interventiongroup [33].

In our study, there was not a difference in the fall rate between the interventiongroup and the control. In a study (Gorski et al., 2017) that assessed the effectiveness of non-pharmacological multi-components prevention in geriatric hospitalized at an internal ward in Poland, the researchers noticed that there was not a statistically significant difference between the intervention and control groups, viewing in the number of falls during hospitalization (4.61% versus 4.61%; *p* = 1.00) [34]. Falls in hospitals represent a major patient-safety problem that may complicate a patient’s care and treatment [35]. It seems that most of the hospitals in Iran could follow fall prevention guidelines and apply some of the strategies of the HELP program.

The results of our study showed that the HELP did not change the length of hospitalization. Similarly, the study on aged care wards reported that multi-component interventions like HELP could not reduce the length of hospital stay [36].

Several factors such as characteristics of patients’ families [37], decision making by the clinical team, family and patients’ conditions, scarcity of equipment and facilities could influence the discharge of elderly patients. Therefore, to determine the effect of the HELP program on the length of hospital stay, our study needs to continue for a longer period and in other hospitals.

Our study has several innovations in comparison with the previous studies. The first one is employing nursing students and family caregivers as volunteers to compensate for the deficit of volunteers in Iran.

In addition,nursing studentsasvolunteers were able to learn special care for elderly patients. On the other hand, the selection of nursing students caused us to spend less time training volunteers, and in fact, the process of selecting volunteers became shorter.

Second, the other studies like Chen et al. [33], have done some HELP protocols, but we did all of the protocols by modifications. The implementation of all interventions was achieved through the proper management of human resources and facilities.

Third, in most of the studies [33, 38], the HELP program has been performed in surgical wards, but we implemented it in the internal wards with patients with various diseases.

The fourthinnovationof our study was the administration of the interventions through using nursing students rather than the existing ward staff. Consequently, the workload of the nurses did not increase in the HELP program. Moreover, since the program was carried out involving nursing students, it was not costly.

The detection of delirium is difficult in routine care [39]. However, our study was standardized using validated and reliable instruments and staff training.

This study had several important limitations. First, due to randomly select the patients in the groups of the study, the effectiveness of HELP interventions results should may not real and accurate (because of differentiation in types of high care need and low needs of care) that might not indicate the real result of the interventions.

The second limitation was the role of family members in the provision of care and support to elderly patients after their admission to the hospital. We provided some interventions in the HELP group with the help of the family members. Similarly, in the control group, also some family caregivers took care of the patients.

## Conclusion

The findings of the present experimental and clinical study reveals that with some modifications to the HELP program, it could be able to decrease delirium in geriatric patients. The HELP may be effective in improving the cognitive and physical functions of elderly patients at moderate to high-risk delirium levels. Therefore, this geriatric interventions program can be successfully applied in internal wards of the hospitals in Iran. It is noteworthy that there is no need for any exclusive facilities and special healthcare services customized for geriatric patients in the research site hospitals and the interventions could be provided by the nursing students and the patient’s caregivers. Hence, the performance of this program did not incur any additional costs for the hospital and the patients.

## Data Availability

The datasets used and/or analyzed during the current study are available from the corresponding author on reasonable request. The full trial protocol is available at https://research.uswr.ac.ir/homepage/loginpage.action.

## Abbreviations

HELP: Hospital Elder Life Program
IG: Intervention group
CG: Control group
CAM: Confusion Assessment Method
SPMSQ: Short Portable Mental StatusQuestionnaire
FGD: Focus Group Discussions
IADL: Instrumental Activity of Daily Living
DS: Digit Span
DSF: Digit Span Forward
DSB: Digit Span Backward
CASH: Canadian Study of Health and Aging
ELS: Elder Life Specialist
MMSE: Mini-Mental State Examination
